# Response of Biological Soil Crust Diazotrophs to Season, Altered Summer Precipitation, and Year-Round Increased Temperature in an Arid Grassland of the Colorado Plateau, USA

**DOI:** 10.3389/fmicb.2012.00358

**Published:** 2012-10-11

**Authors:** Chris M. Yeager, Cheryl R. Kuske, Travis D. Carney, Shannon L. Johnson, Lawrence O. Ticknor, Jayne Belnap

**Affiliations:** ^1^Bioscience Division, Los Alamos National LaboratoryLos Alamos, NM, USA; ^2^Computer, Computational, and Statistical Sciences Division, Los Alamos National LaboratoryLos Alamos, NM, USA; ^3^U.S. Geological Survey, Southwest Biological Science CenterMoab, UT, USA

**Keywords:** biological soil crust, *nifH*, cyanobacteria, climate change, semi-arid, altered precipitation, nitrogen fixation, *Scytonema*

## Abstract

Biological soil crusts (biocrusts), which supply significant amounts of fixed nitrogen into terrestrial ecosystems worldwide (∼33 Tg y^−1^), are likely to respond to changes in temperature and precipitation associated with climate change. Using *nifH* gene-based surveys, we explored variation in the diazotrophic community of biocrusts of the Colorado Plateau, USA in response to season (autumn vs. spring), as well as field manipulations that increased the frequency of small volume precipitation events and year-round soil temperature. Abundance of *nifH* genes in biocrusts ranged from 3 × 10^6^ to 1 × 10^8^ g^−1^ soil, and *nifH* from heterocystous cyanobacteria closely related to *Scytonema hyalinum*, *Spirirestis rafaelensis*, and *Nostoc commune* comprised >98% of the total. Although there was no apparent seasonal effect on total *nifH* gene abundance in the biocrusts, T-RFLP analysis revealed a strong seasonal pattern in *nifH* composition. *Spirirestis* *nifH* abundance was estimated to oscillate 1 to >2 orders of magnitude between autumn (low) and spring (high). A year-round increase of soil temperature (2–3°C) had little effect on the diazotroph community structure over 2 years. Altered summer precipitation had little impact on diazotroph community structure over the first 1.5 years of the study, when natural background patterns across years and seasons superseded any treatment effects. However, after the second summer of treatments, *nifH* abundance was 2.6-fold lower in biocrusts receiving altered precipitation. Heterocystous cyanobacteria were apparently more resilient to altered precipitation than other cyanobacteria. The results demonstrate that diazotrophic community composition of biocrusts in this semi-arid grassland undergoes strong seasonal shifts and that the abundance of its dominant members decreased in response to more frequent, small volume precipitation events.

## Introduction

In many arid and semi-arid regions, nitrogen (N) is considered second only to water as the resource that constrains ecosystem productivity (Peterjohn and Schlesinger, 1990; Hooper and Johnson, 1999; Yahdjian et al., 2011; Ladwig et al., 2012). In these plant-sparse ecosystems, complex communities of cyanobacteria and other bacteria, algae, fungi, lichens, and mosses form biological crusts (hereafter referred to as biocrusts) on the soil surface. Biocrusts play an essential role in soil stability and albedo, water infiltration, seed germination, and carbon (C) and N inputs (Belnap and Lange, 2003). Globally, soil biocrusts are estimated to be responsible for ∼30% of biologically fixed N in terrestrial ecosystems or ∼33 Tg per year; (Elbert et al., 2012). Estimates of annual N inputs from biocrusts of the cool desert ecosystem of the Colorado Plateau, USA, range between 0.7 and 13 kg N ha^−1^ (Jeffries et al., 1992; Belnap, 2002) which equals or well exceeds the rate of atmospheric N deposition estimated for this region (0.5–4.0 kg N ha^−1^; Pardo et al., 2011). Indeed, N content and isotope fractionation (δ^15^N) measurements in a pinyon-juniper woodland of the Colorado Plateau indicated that the primary source of N for this ecosystem was biocrust N_2_ fixation (Evans and Ehleringer, 1993).

The *nifH* gene encodes the reductase subunit of the enzyme responsible for all biological N_2_ fixation, nitrogenase, and has been used extensively as a molecular marker to identify diazotrophic members of natural communities (Zehr et al., 2003; Gaby and Buckley, 2011). Molecular surveys of *nifH* composition and relative abundance, paired with culturing, microscopy, and field measurements of nitrogenase activity indicate that biological N_2_ fixation in biocrusts of the Colorado Plateau is primarily carried out by three genera of heterocystous cyanobacteria, *Nostoc*, *Spirirestis*, and *Scytonema* (Belnap, 2002; Yeager et al., 2007). *Nostoc* and *Scytonema* spp. are well-known inhabitants of biocrusts worldwide and species from both genera are known to survive under extremes of temperature, desiccation, and UV stress, allowing them to colonize some of the harshest terrestrial environments on Earth (Potts, 2000; Péli et al., 2011). In biocrusts, *Nostoc* and *Scytonema* spp. are present as free-living organisms and epiphytes on bryophytes and lichens. Additionally, *Nostoc* is a common lichen photobiont (i.e., *Collema*, *Peltula*, and *Heppia*; Veluci et al., 2006; Lücking et al., 2009). Both of these genera are easily identified by microscopic analysis. On the other hand, *Spirirestis* is a recently described genus that is much more difficult to identify in biocrust samples and is difficult to morphologically distinguish from other heterocystous species (e.g., *Tolypothrix*). Little is known about *Spirirestis*’ geographical distribution, response to extreme conditions, or function in biocrust communities (Flechtner et al., 2002).

Rates of N_2_ fixation in top soils from the Colorado Plateau have been shown to peak below the biocrust surface (∼7 mm), especially when heterocystous cyanobacteria are relatively scarce, and it has been suggested that non-cyanobacterial, heterotrophic diazotrophs may play an important role in N_2_ fixation below the crust surface (Johnson et al., 2005). Cluster O sequences, which putatively represent alphaproteobacterial *nifH* sequences, have been detected in all *nifH* surveys of Colorado Plateau soils conducted by our group, and typically comprise 5–15% of the total *nifH* pool of biocrusts (e.g., Yeager et al., 2004). However, neither the composition nor the population size of the sub-biocrust diazotrophic community has been assessed.

Climate models generally predict a warmer (up to 6°C increase) Colorado Plateau over the next 30–100 years (Christensen et al., 2004; Cayan et al., 2010). Precipitation patterns are also predicted to change, but the direction and magnitude of this change remains uncertain (Weltzin et al., 2003; Solomon et al., 2007). One of the likely outcomes is more frequent summer precipitation events, but with negligible change in total summer precipitation (i.e., more frequent, smaller volume events; Weltzin et al., 2003). In October 2005 a long-term field study in the Castle Valley area of the Colorado Plateau was initiated to investigate the impact of increased temperature (2–3°C) and/or increased frequency of small volume (≤1.2 mm) summer precipitation events on biocrust composition and function, soil nutrient cycling, and plant productivity in this semi-arid grassland ecosystem. From this field study we recently demonstrated that an increase in the frequency of small volume, summer precipitation events led to mass mortality of biocrust mosses and cyanobacteria within 1–2 years (Johnson et al., 2012; Reed et al., 2012; Zelikova et al., 2012). We present here the influence of season and year on the abundance and composition of the diazotrophic bacteria in biocrusts, and in soils 5 cm beneath the biocrust. We examine the possibility that distinct diazotrophic bacteria subsist in a distinct zone of the soil profile, 5 cm below the surface (the top 1 cm of the topsoil were defined as the biocrusts in this study). The response of biocrust diazotrophs to a 2–3°C increase in soil temperature and an altered summer precipitation regime, consistent with predicted climate change scenarios for this region, were also monitored over a 2 year period.

## Materials and Methods

### Field site description and experimental design

The study site is located on the Upper Colorado Plateau and is located near Castle Valley, UT, USA (38.67485 N-109.4163 W, 1310 m above sea level). The region is classified as a cool desert ecosystem, and the site is within a semi-arid grassland comprised primarily of two perennial grasses, *Pleuraphis jamesii* (syn. *Hilaria jamesii*) and *Achnatherum hymenoides* (syn. *Stipa hymenoides*), as well as the shrub, *Atriplex confertifolia*. The soil surface in plant interspaces is covered by a biocrust comprised of cyanobacteria, moss (*Syntrichia caninervis*), and lichen (*Collema tenax* and *Collema coccophorum*). Soil characteristics and greater detail concerning the study site are described by Zelikova et al. (2012) and Johnson et al. (2012).

A randomized block design was established consisting of 20 2 m × 2.5 m plots with five field replicates for each of the following three treatments and single control: (1) increased year-round temperature with infrared lamps (IR), (2) altered summer precipitation (W), (3) a combination of increased temperature and altered summer precipitation (IRW), and (4) a control plot outfitted with a non-functional IR lamp (LC). An additional control (C) consisted of undisturbed biocrusts collected adjacent to our study area. Warming treatments were designed to increase soil temperatures 2°C at 2 cm soil depth and were achieved with 800 W IRs situated 1.3 m above the soil surface. The field site came on-line October 2005 and the IR lamps warmed surface soils 2°C 24 h day^−1^. The precipitation regime (hereafter referred to as FSVP, or Frequent Small Volume Precipitation) was designed to provide more frequent, small volume wetting events (4× the historical average of ≤1.2 mm summer rainfall events) during the summer. To achieve this, 2 mm watering treatments were performed 2–3× weekly from mid-June to mid-September 2006 and 2007. Rainout shelters were not established, thus natural precipitation occurred on all plots. In aggregate, the W and IR&W plots received ∼50% more total precipitation volume than the historical average.

### Soil collection, DNA extraction, and scytonemin quantification

Soil samples and DNA extracts are the same as those reported in Johnson et al. (2012). Briefly, soil samples were collected for DNA analysis in the spring (May) and autumn (October/September) from October 2005 through September 2007. Three soil samples were collected from three of the five blocks of each treatment and the controls (nine field samples for each treatment and control at each time point). From each plot, soil was collected from two depths: (1) biocrust material (∼5 g) was collected from a 2 cm × 2 cm area to ≤1 cm depth and (2) sub-biocrust soil (∼5 g) was collected at 5 cm below the surface with a 2.5 cm soil corer. Soil samples were immediately placed on dry ice for transport to the laboratory, whereupon they were stored at −80°C. DNA was extracted from soil samples using the FastDNA Spin Kit for Soil (MP Biomedicals) following the manufacturers instructions and stored at −70°C. Biocrusts and sub-crust soils were dry at each sampling event as determined by feel and visualization. The Kuske and Belnap labs have each measured dry weights of visually and texturally dry biocrusts and soils of the region on multiple occasions and found that moisture content is negligible (<0.5%).

Pigment extraction and analysis was performed by Zelikova et al. (2012). Briefly, for pigment analysis five soil samples were collected from the upper 0.5 cm of the biocrust surface from each of the five blocks of the treatments and control plots. The samples were immediately transported to the lab where they were passed through a 2 mm sieve and ground with mortar and pestle. Pigments were acetone-extracted from biocrusts, filtered, and analyzed via HPLC with a photodiode detector at 436 nm as previously described (Karsten and Garcia-Pichel, 1996). A modified extinction coefficient of 60.8 L g^−1^ cm^−1^ at 436 nm was used to determine scytonemin concentrations (μg g^−1^ soil; Zelikova et al., 2012).

### Standard *nifH* PCR, cloning, and sequence analysis

A portion of the *nifH* gene (∼360 bp) was amplified from soil DNA extracts using a nested reaction modified from Yeager et al. (2004). The first reaction amplified an ∼1000 bp fragment using primers 19F (5′-GCIWTYTAYGGIAARGGIGG; Ueda et al., 1995) and nifH3 (5′-ATRTTRTTNGCNGCRTA; Zani et al., 2000). Each 15 μL reaction was carried out in triplicate and contained the following: 1× PCR buffer I (Applied Biosystems), 9.6 pmol each primer, 0.8 mM dNTPs, 3 μg BSA, 20 mM trehalose (Sigma-Aldrich), 0.75 U Ampli-Taq LD Polymerase (Applied Biosystems), and 1 μL template DNA. The thermal cycle profile consisted of: 95°C for 5 min; 23 cycles of 48°C for 1 min, 72°C for 1 min, and 94°C for 45 s; 72°C final extension step for 10 min. For the second PCR of the nested method, primers nifH11 (5′-GAYCCNAARGCNGACTC) and nifH22 (5′-ADWGCCATCATYTCRCC) were used to amplify a ∼360 bp *nifH* fragment using 1 μL of a 1:10 dilution of the first amplification reaction as template. Reaction mixtures (50 μL) contained the same concentrations of reagents and primers as described above. Thermal cycling conditions were also the same, except that the annealing temperature was raised to 55°C and the number of cycles was increased to 30 cycles.

Amplicons were pooled and gel purified (QIAquick) and cloned using the TOPO-TA pCR2.1 kit (Invitrogen) according to the manufacturer’s directions. Clones were sequenced bidirectionally using the M13 primers and Sanger Technology at the LANL JGI. Sequences were manually trimmed, edited, and assembled in Sequencher (GeneCodes) and then cut *in silico* to determine the expected peak size.

### Quantitative PCR of the *nifH* gene

DNA extracts were normalized to equal concentrations (25 ng μL^−1^ for crust samples and 12.5 ng μL^−1^ for sub-biocrust samples) and diluted either 1:10 or 1:100 in nuclease free water, before using as template for Quantitative PCR (qPCR). Each 30 μL qPCR reaction contained 15 μL iQ SYBR Green Supermix (Bio-Rad), 15 pmol of each primer (CY81F, 5′-GYGCTGTNGAAGATATWGAAC; CY226R, 5′-GCCGTTTTCTTCCAAGAAGTT), and 1 μL of template to amplify a ∼145 bp fragment of the *nifH* gene (Yeager et al., 2004). Reactions were carried out in triplicate using a Bio-Rad MyiQ thermal cycler (Bio-Rad Laboratories) with experimental plate well factors. The thermal profile included an initial denaturation step (95°C) for 7 min, 40 cycles of amplification (95°C for 30 s, 53°C for 25 s, 72°C for 30 s). Each run included a melt curve analysis (80 cycles beginning at 55°C, increasing 0.5°C every 2 cycles) to ensure that a single product was produced. *In silico* analysis of the 502 *nifH* clones sequenced in this study revealed that CY226R was complimentary to >99% of the *nifH* sequences and CY81F was complimentary to >90% of the sequences (>99%, if base mismatches at the second position from the 5′ end of the primer, which should have little effect on primer binding, are discounted).

To generate a standard curve for the *nifH* qPCR, 12 clones were randomly selected from the biocrust *nifH* clone library (described above) and grown overnight in selective LB medium. The cultures were pooled and plasmid DNA was extracted using the QIAprep Spin Miniprep Kit (QIAGEN). Plasmid DNA was quantified using PicoGreen (Molecular Probes) and linearized using *Sca*I (New England Biolabs). Standard curves were constructed using a 10-fold dilution series (1 × 10^1^ to 1 × 10^9^ *nifH* copies per reaction) of linearized plasmid DNA harboring the *nifH* fragments. The PCR efficiency determined from the standard curve ranged from 90 to 100%.

### T-RFLP analysis

Nested PCR to generate *nifH* for terminal restriction fragment (TRF) length polymorphism analysis (T-RFLP) were carried out as described above for cloning and sequencing, except that the nifH11 primer was 5′-labeled with 6-fluorescein. Triplicate reactions were performed for each sample, combined, and purified using a 1% agarose gel and the Qiaquick PCR Purification Kit (QIAGEN). Purified PCR products were quantified using PicoGreen dsDNA Quantitation Reagent (Molecular Probes) and digested (50 ng) for 4.5 h at 55°C with 2.5 U *Mae*III (Roche Applied Science). Restriction digests were desalted and concentrated by ethanol precipitation, then suspended in 25 μL of formamide. T-RFLP analysis of the 6-fluorescein-labeled *nifH* fragments was performed with a 3130xL genetic analyzer, and the data were analyzed using GeneMapper software (Applied Biosystems).

Restriction fragment peak areas (integrated fluorescence) were converted to a percentage of the total peak area (total fluorescence) for each sample. Peaks that represented <0.5% of the total peak area for a sample were precluded from analysis. In some T-RFLP profiles, 330 and 356 bp TRF peaks were detected. Based on analysis of extensive *nifH* clone libraries (1000’s of *nifH* sequences) derived from biocrusts at this study site and nearby areas, it was determined that these peaks represented uncut and partially digested fragments. In cases where these uncut/partially digested peaks comprised >25% of the total T-RFLP profile (peak area basis), the entire profile was rejected (34 out of 210, or 16%, of the T-RFLP profiles were removed). For the remaining profiles (*n* = 176), all 330 and 356 bp peaks were removed and the remaining TRFs were normalized to percentages.

### Statistical analyses

Statistical analyses were implemented using the JMP software package (v5.1, SAS). After preliminary analyses found no significant block effect in the qPCR or sequence datasets, the data were analyzed as nine replicate samples for each soil depth, in each treatment, and for each time point. Comparison of *nifH* gene copy number (qPCR data) between the control biocrust and 5 cm deep soil samples was conducted on samples pooled across all time points, using ANOVA followed by a two-way pairwise *t*-test. Comparison of nifH gene copy number (qPCR data) across the five sampling points, in either biocrusts or in sub-biocrust soils, was conducted using ANOVA followed by Tukey’s HSD mean separation procedure. Analysis of temperature and FSVP effects on *nifH* gene copy number (qPCR data) and scytonemin/chlorophyll *a* ratios were conducted separately at each sampling time point, and were compared using ANOVA with a factorial model (IR treatment, W treatment, IR × W interaction). The total experimental ANOVA *P* value, and the *P* value for significant variables are presented in the results, tables, and/or table legends. The normalized (percent of total) T-RFLP datasets were non-parametric, and comparisons of the relative representation of *Scytonema*, *Spirirestis*, and *Nostoc* with season and among experimental treatments were conducted using ANOVA followed by a Wilcoxon/Kruskal–Wallace test. Similarities among the composition of T-RFLP patterns were measured using a Bray–Curtis distance measure based on the relative abundance of each of the three major genera, and visualized using agglomerative hierarchical clustering, using the R software program (version 2.11.1; www.r-project.org).

## Results

### Biannual quantification (qPCR) of *nifH* sequences in control biocrust and sub-biocrust soils

Comparison of diazotroph abundance, using *nifH* gene copy number in undisturbed biocrusts and sub-biocrust soils was conducted on data pooled across all the sampling times (*n* = 87–88 per soil type). A pairwise *t*-test (*P* < 0.000) indicated that biocrusts contained significantly more (average of 55 times more) *nifH* gene copies than the sub-biocrust soils 5 cm below the surface (Table 1). On average, there was 3.6 × 10^7^ and 6.5 × 10^5^ *nifH* copies g^−1^ soil in the biocrust and sub-biocrust soil, respectively. These values are within, or slightly above, the range of *nifH* copy number typically found in soils (1 × 10^5^ to 1 × 10^7^ copies g^−1^ soil; Pereira e Silva et al., 2011 FEMS), and align closely with qPCR enumeration of *nifH* in mature biocrusts (∼2 × 10^7^ *nifH* copies g^−1^ soil) of a nearby region of the Colorado Plateau (Yeager et al., 2004).

**Table 1:** **Abundance of *nifH* in undisturbed biocrusts and sub-biocrust soils**.

Date	*nifH* copies g^−1^ soil^a^	
	Biocrust	Sub-biocrust
October 2005	4.9 × 10^6^ ± 9.4 × 10^5^ c	9.5 × 10^5^ ± 2.7 × 10^5^ a,b
May 2006	2.0 × 10^7^ ± 3.3 × 10^6^ b,c	1.7 × 10^5^ ± 6.6 × 10^4^ b
September 2006	3.4 × 10^7^ ± 5.7 × 10^6^ b	3.0 × 10^5^ ± 1.1 × 10^5^ b
May 2007	6.2 × 10^7^ ± 6.3 × 10^6^ a	3.7 × 10^4^ ± 1.7 × 10^4^ b
September 2007	6.0 × 10^7^ ± 9.9 × 10^6^ a	1.8 × 10^6^ ± 6.1 × 10^5^ a

*^a^Values represent the mean and standard error (*n* = 17–18). One way ANOVA followed by Tukey’s HSD mean separation procedure was performed for biocrust and sub-biocrust samples across sampling dates (biocrust, *P* < 0.000; sub-biocrust, *P* = 0.001). Values comparing sampling dates (within a column) followed by the same letter were not significantly different*.

The number of *nifH* copies in the biocrust steadily increased from October 2005 to May 2007 (ANOVA *P* < 0.000, Tukey HSD mean separation results in Table 1); whereas, the abundance of *nifH* in the sub-biocrust soils exhibited considerable variability between sampling dates (ANOVA *P* < 0.000, Tukey HSD mean separation results; Table 1) and did not exhibit a year-over-year trend. A consistent correlation between the *nifH* abundance in the biocrust and sub-biocrust soils based on season was not detected. A seasonal effect on *nifH* copy number in the biocrusts was not detected, but *nifH* abundance in the sub-biocrust soil was significantly greater in autumn than spring (*t*-test, *P* = 0.004). Sub-biocrust *nifH* copy number averaged 1.1 × 10^5^ in spring (*n* = 34) and approximately an order of magnitude more in autumn (1.0 × 10^6^; *n* = 53).

### Quantification (qPCR) of *nifH* sequences in biocrusts subjected to increased temperature and FSVP

**Figure 1:**
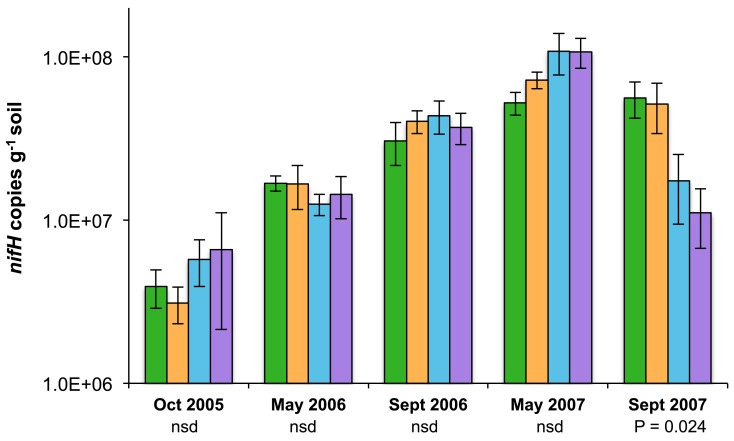
**Quantification of *nifH* (copies g^−1^ soil) biannually for 2 years in biocrusts from four different treatment plots: non-functional IR lamp controls (LC; green bars, left-most in each set), increased temperature year-round (IR; orange bars), FSVP (W; blue bars), and both FSVP and increased year-round temperature (IR&W; purple bars, right-most in each set)**. Bars show the mean and standard error (*n* = 9; except October 2005 IR&W where *n* = 3 and May 2007 LC where *n* = 5). ANOVA *F*-test results (Prob > F) are displayed immediately below the date on the horizontal axis. Biocrusts treated with FSVP (W and IR&W) were significantly (ANOVA *t*-test, *P* = 0.003) different from biocrusts that received ambient precipitation (LC, IR) in September 2007.

Between October 2005 and May 2007, *nifH* abundance in each of the manipulated plots increased approximately one order of magnitude, mirroring the increase observed in the control plot biocrusts (Figure 1). By September 2007, biocrust *nifH* abundance was markedly reduced in both plots that received FSVP (W, IR&W) compared to the biocrusts that received just ambient precipitation (LC, IR) (ANOVA Prob > F = 0.024; effect of watering *t*-test 0.003). At this time, FSVP-treated biocrusts (W, IR&W) exhibited 2.6-fold fewer *nifH* copies g^−1^ soil than biocrusts receiving ambient precipitation (LC, IR). Increased year-round temperatures (IR treatments) did not significantly affect biocrust *nifH* abundance for any sampling date.

**Figure 2:**
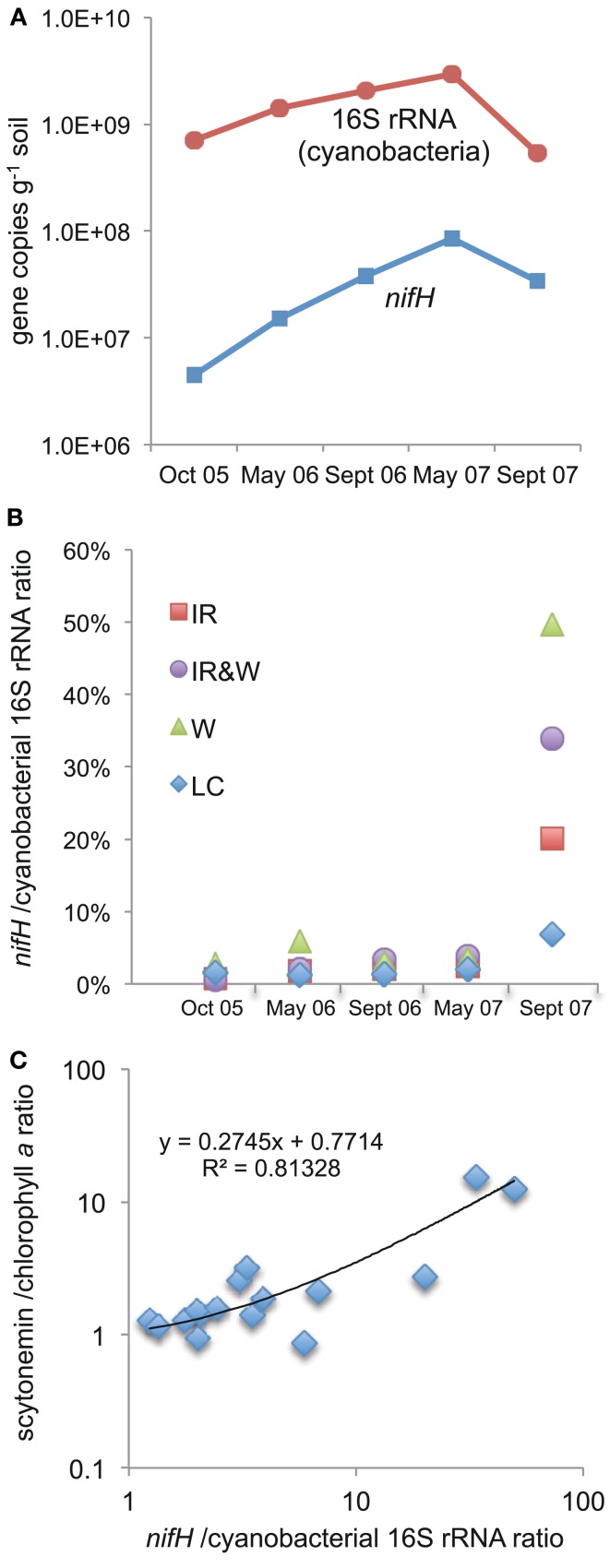
**The relative abundance of heterocystous cyanobacteria in biocrusts as a fraction of the total cyanobacterial population**. **(A)** Abundance of *nifH* and cyanobacterial 16S rRNA genes averaged across all treatments over the course of the study, **(B)** ratio of *nifH*:cyanobacterial 16S rRNA genes in biocrusts as a function of treatment over time (treatment designations are defined in the legend for Figure 1), and **(C)** correlation of two independent measures of the relative proportion of heterocystous cyanobacteria in the total biocrust cyanobacterial population (scytonemin:chlorophyll *a* ratio and *nifH*:cyanobacterial 16S rRNA ratio).

The cyanobacterial 16S rRNA gene copy number in these samples was determined by Johnson et al. (2012) and is shown in Figure 2A. The ratio of *nifH* to cyanobacterial 16S rRNA gene copies in the biocrust samples was determined, and by assuming a 1:1 ratio of *nifH* and 16S rRNA gene copies per cyanobacterial cell, it was determined that diazotrophic, heterocystous cyanobacteria comprised ∼0.5–6% of the total cyanobacterial population across each treatment from October 2005 to May 2007 with no apparent trends associated with season or treatment (Figure 2B). These values are consistent with the results from our recent 16S rRNA gene pyrosequencing surveys, where we found that *Nostoc*, *Scytonema*, and *Spirirestis* sequences comprised ∼3% of all cyanobacterial sequences in undisturbed biocrusts from this study site (unpublished results). In September 2007, the LC biocrust cyanobacterial community consisted of 7% heterocystous cyanobacteria, but was much lower than the treated plots where the percentage of heterocystous species comprising the total cyanobacterial population had increased to 20% in IR-treated biocrusts, 34% in IR&W-treated biocrusts, and 50% in W-treated biocrusts (Figure 2B). Regression analysis between the *nifH*/cyanobacterial 16S rRNA ratio and the scytonemin/chlorophyll *a* ratio (see next section) showed that the two methods provided congruent results (Figure 2C, *R*^2^ = 0.81). Thus, multiple approaches allowed us to determine *relative* changes in the fraction of heterocystous cyanobacteria comprising the total cyanobacterial population in response to perturbation.

### Biannual quantification of scytonemin in biocrusts subjected to increased temperature and FSVP

**Figure 3:**
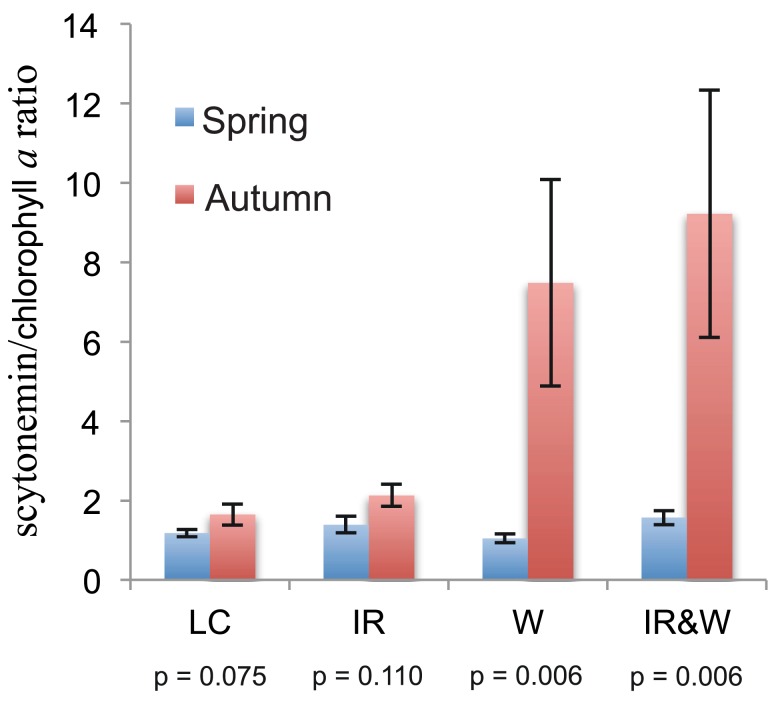
**Ratio of scytonemin to chlorophyll *a* in biocrusts subjected to increased year-round temperature, FSVP, or both**. Bars show the mean and standard error of the scytonemin to chlorophyll *a* ratio for biocrusts collected in spring (May 2006 and 2007 pooled, *n* = 15) and autumn (October 2006 and September 2007 pooled, *n* = 10) for each treatment (treatment designations are defined in the legend for Figure 1). The results of pairwise *t*-tests between seasons within each treatment are shown below each pair of bars.

Scytonemin is produced primarily by heterocystous cyanobacteria in the Colorado Plateau biocrusts and has been used as an indirect measure of the biomass/physiological status of these microorganisms (Bowker et al., 2002). Soil scytonemin concentrations were determined biannually from May 2006 to September 2007 and ranged from 0.5 to 1.8 μg g^−1^ soil, but neither seasonal nor treatment effects were observed (data not shown). However, ratios of scytonemin to chlorophyll *a* were significantly impacted by FSVP. The scytonemin/chlorophyll *a* ratio was 2.1- and 5.7-fold greater in the biocrusts treated with FSVP (W, IR&W) than biocrusts that received ambient precipitation (LC, IR) in September 2006 and 2007, respectively (Table 2). This trend was not evident in biocrusts sampled in May 2006 or 2007. Indeed, when the data were pooled by season (May 2006 + May 2007 is spring, *n* = 15 observations vs. October 2006 + September 2007 is autumn, *n* = 10 observations), the scytonemin/chlorophyll *a* ratio was consistently higher (pairwise *t*-test, *P* < 0.05) in autumn samples than those collected in spring, regardless of treatment (data not shown). However, treatment comparisons using a factorial model were statistically significant only in the two September sampling dates (Table 2), and the only significant variable was FSVP (W and IR&W bars in Figure 3). In the LC and IR-treated biocrusts, the ratio of scytonemin/chlorophyll *a* was 1.4- to 1.5-fold higher in the autumn vs. spring, whereas in the W- and IR&W-treated biocrusts the ratios were 7.2- and 5.9-fold higher, respectively, in autumn.

**Table 2:** **The ratio of scytonemin to chlorophyll *a* in biocrusts across treatments**.

Treatment^a^	Scytonemin/chlorophyll *a* ratio^b^			
	May 2006	September 2006	May 2007	September 2007
LC	1.29 ± 0.33	1.16 ± 0.32	0.95 ± 0.33	2.13 ± 1.01
IR	1.29 ± 0.84	1.51 ± 0.95	1.58 ± 0.73	2.73 ± 1.65
W	0.86 ± 0.24	2.57 ± 0.81	1.40 ± 0.51	12.39 ± 9.56
IR&W	1.42 ± 0.54	3.17 ± 0.93	1.87 ± 0.94	15.27 ± 11.27
	nsd	*P* = 0.004	nsd	*P* = 0.026

*^a^Treatment designations are given in the legend for Figure 1*.

*^b^Scytonemin/chlorophyll *a* ratio is presented as the mean ± SD (*n* = 5 for all values, except May 2006 where *n* = 10). Experimental ANOVA *F*-test results using a factorial model are displayed beneath each sampling date, showing significant treatment differences in September 2006 and 2007. Only the FSVP variable was significant (September 2006 ANOVA *t*-test for FSVP, *P* = 0.000; and September 2007 ANOVA *t*-test, *P* = 0.004). Thus, FSVP-treated biocrusts exhibited higher ratios than non-watered biocrusts in autumn*.

The FSVP treatments were applied to the W and IR&W plots two to three times per week from mid-June until mid-September (2006 and 2007), while all plots received ambient precipitation from September through June. Thus, September samples were collected immediately following the FSVP treatment regime, whereas all biocrusts collected in May had received the same precipitation (ambient) over the previous 8 months.

### Sequence analysis of *nifH* clone libraries from biocrust and sub-biocrust soils

**Figure A1:**
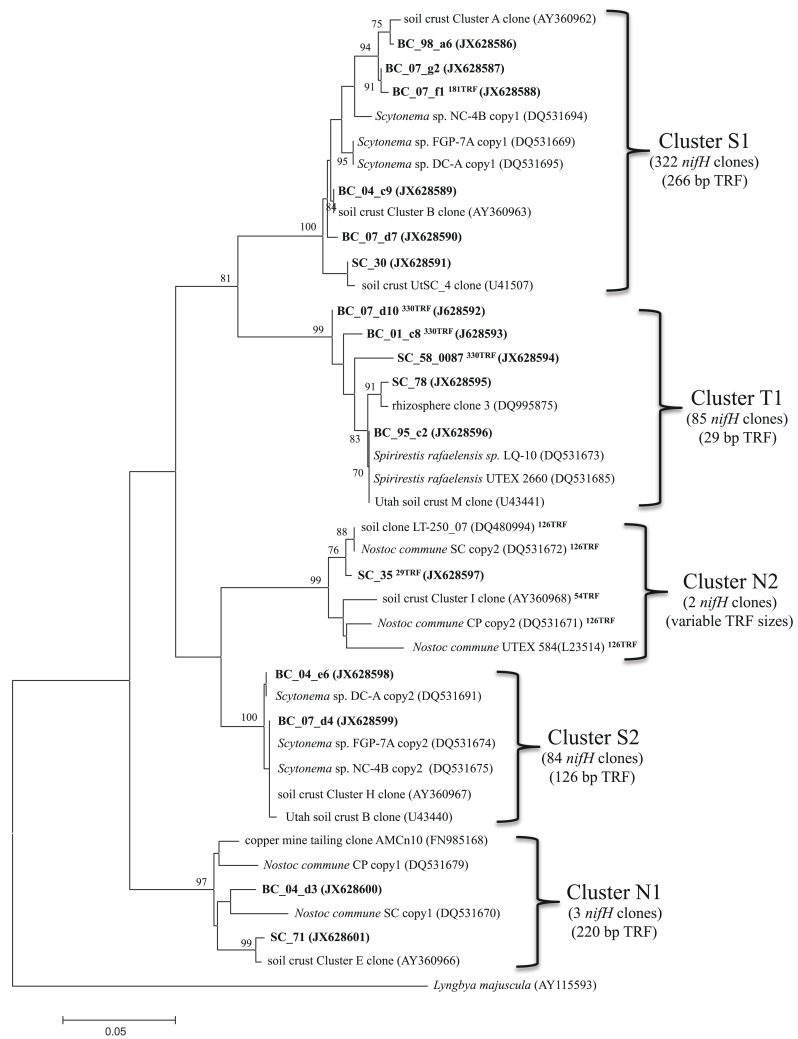
**Neighbor-joining tree containing representative, partial *nifH* sequences (310 bp) found in this study (in bold; SC clones were from sub-biocrust soils and BC clones from biocrusts)**. Sequence clusters corresponding to *nifH* sequences from *Scytonema* (S1, S2), *Spirirestis* (T1), and *Nostoc* spp. (N1, N2) are designated to the right of the tree. The TRF size of the majority of *nifH* sequences within each cluster is shown below each cluster name. Exceptions are indicated by superscript TRF values assigned to the corresponding taxon name. For example, BC_07_d10 yielded a 330 bp TRF whereas the majority of sequences in the T1 cluster yielded a TRF size of 29 bp (see the Table 3 legend for a complete accounting of TRF variants in each cluster). The number of sequences within each *nifH* cluster identified in this study is also shown in parenthesis. GenBank accession numbers for sequences are in parenthesis following the clone or taxon name.

Clone libraries of *nifH* sequences were generated from biocrust (*n* = 5 libraries, 390 sequences) and sub-biocrust soils (*n* = 2 libraries, 119 sequences). All *nifH* sequences were similar to those previously identified in biocrusts of the Colorado Plateau and could be grouped into seven clusters with intra-cluster mean distances ranging from 0 to 0.030 (Table 3; Figure A1 in Appendix). Clusters S1A and S1B are closely related sequence types (between cluster mean distance of 0.030) and can be classified as *nifH* copy #1 from the cyanobacterium *Scytonema hyalinum* and related species (Yeager et al., 2007). Cluster S2 sequences correspond to *nifH* copy #2 from *S. hyalinum*. The *S. hyalinum* sequences comprised 88 and 59% of the total *nifH* clones analyzed from the biocrust and sub-biocrust soils, respectively. The second most abundant diazotroph identified in the *nifH* clone library survey was *Spirirestis rafaelensis*. Cluster T1 sequences shared 98–100% similarity with the single *nifH* copy that has been identified in *S. rafaelensis* (Yeager et al., 2007), and comprised 10 and 38% of the total *nifH* sequence pool from the biocrust and sub-biocrust soils, respectively. A small percentage of the *nifH* sequences (≤2%), designated Clusters N1 and N2, could be classified as *nifH* copy #1 and #2, respectively, from *Nostoc commune* and related species.

**Table 3:** **Biocrust and sub-biocrust *nifH* sequence types from clone libraries**.

*nifH* cluster	Intra-cluster mean distance	Taxon^a^	*nifH* distribution^b^		TRF size (bp)^c^
			Biocrust	Sub-biocrust	
S1A	0.006	*S. hyalinum* NCC-4B (DQ531694; 97–100%)	115	42	266
S1B	0.009	*S. hyalinum* DC-A (DQ531695; 97–99%)	152	13	266
S2	0.006	*S. hyalinum* DC-A (DQ531691; 99–100%)	69	15	126
N1	0.030	*N. commune* MFG-1 (DQ531687; 97–98%)	1	2	250
N2	0	*N. commune* SC (DQ531672; 99%)	0	2	Variable^d^
T1	0.007	*S. rafaelensis* UTEX2660 (DQ531685; 98–100%)	40	45	29
O	0.001	α-Proteobacteria	6	0	120

*^a^Taxonomic assignment of the *nifH* sequence clusters. The accession number of the most closely related *nifH* sequence from a cultured strain is shown in parenthesis followed by the percent similarity between that *nifH* sequence and those in the cluster*.

*^b^Number of clones within each *nifH* sequence cluster originating from the biocrust or sub-biocrust soils*.

*^c^TRF size was determined by *in silico* digestion of all sequences comprising each cluster. Three of the clusters contained a small fraction of sequences that yielded TRF sizes different from the values listed in Table 3 (cluster S1A, 1/168 sequences yielded a 29 bp TRF; cluster S1B, 1/176 sequences yielded a 181 bp TRF; cluster T1, 4/84 sequences yielded a 330 bp TRF)*.

*^d^Sequences that fall within the N2 Cluster yield TRFs of varying size (29, 54, 126; see Figure A1 in Appendix). The two sub-crust sequences identified in this study each yielded a 29 bp TRF when cut *in silico* with *Mae*III*.

The only non-cyanobacterial *nifH* sequences identified in this study consisted of a single type, Cluster O, and were obtained from just one biocrust library. Cluster O sequences comprised 1.5% of the total biocrust *nifH* pool and likely derive from an α-proteobacteria species (88–89% similarity to *nifH* sequences from *Rhodopseudomonas palustris*, *Gluconacetobacter diazotrophicus*, and *Azospirillum brasilense*).

*In silico* digestion (targeting the *Mae*III cut site) was performed on all *nifH* sequences from the clone libraries in order to link individual TRFs to the *nifH* sequence types (clusters) designated in Table 3. From this analysis it was established that the 126 and 266 bp TRFs represented *nifH* sequences from the *Scytonema* cluster, the 29 bp TRF represented *nifH* sequences from the *Spirirestis* cluster, the 54 and 250 bp TRFs represented the *Nostoc nifH* cluster, and the 120 bp TRF represented the α-proteobacteria-like *nifH* sequences of Cluster O [Cluster O comprised <0.5% of the total T-RFLP profile in all biocrust and sub-biocrust samples analyzed (data not shown)].

### Biannual T-RFLP analysis of *nifH* composition and relative abundance in biocrusts and sub-biocrust soils

T-RFLP analysis of *nifH* amplicons was performed to provide an evaluation of the composition and relative abundance of diazotrophic taxa in biocrust and sub-biocrust samples collected biannually (spring and autumn) over 2 years. Agglomerative hierarchical clustering of Bray–Curtis distances derived from normalized *nifH* T-RFLP patterns revealed a seasonal distinction in the diazotrophic community composition of undisturbed biocrusts, but not in sub-biocrust samples at our study site (data not shown). In the undisturbed sub-biocrust samples, *Scytonema* peaks comprised 81% of the total *nifH* T-RFLP profile in the spring and 84% in the autumn, *Spirirestis* peaks comprised 18% of the *nifH* T-RFLP profile in the spring and 15% in the autumn, and *Nostoc* peaks comprised 1% of the *nifH* T-RFLP profile in both the spring and autumn (data not shown).

**Figure 4:**
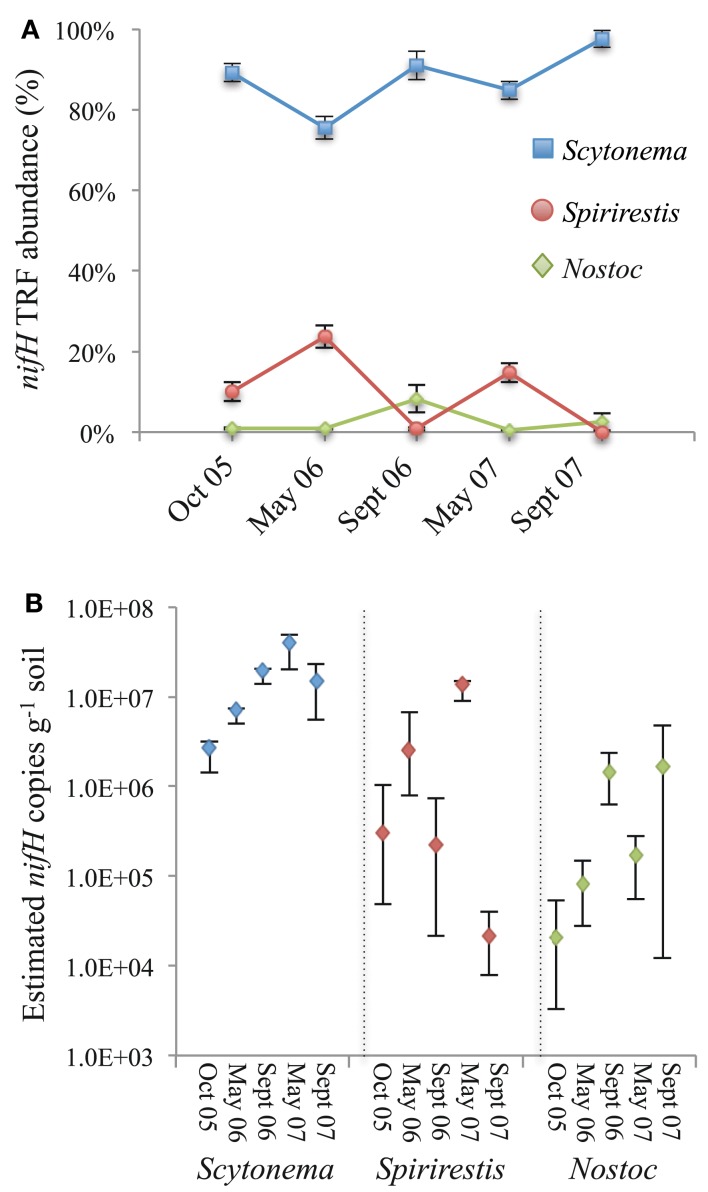
**(A)** Seasonal shifts in the relative abundance of *Scytonema* (blue squares), *Spirirestis* (red circles), and *Nostoc* spp. (green diamonds) *nifH* sequences in biocrusts from October 2005 to September 2007. Data are means (±1 SE) of T-RFLP relative abundance values from 26 to 36 biocrust replicates across all treatments. **(B)** Estimated *nifH* copies g^−1^ soil across seasons for *Scytonema*, *Spirirestis*, and *Nostoc* spp. Copies of *nifH* for each genera per g^−1^ of biocrust was estimated by multiplying the total *nifH* abundance g^−1^ soil (determined by qPCR) by the percent relative abundance of each genera-specific *nifH* sequence type (determined by T-RFLP). Data are shown as the mean (diamonds) and range of values for all treatments.

In the majority (93%) of biocrust samples analyzed across all time points and treatments, TRFs representing *Scytonema* and *Spirirestis*, filamentous strains that can wind contiguously through the upper millimeter of the biocrust, comprised over 95% of the diazotrophic community (Figure 4A). Among these two genera *nifH* TRFs assigned to *Scytonema* were particularly dominant, comprising 75–99% of the T-RFLP profiles.

For statistical analyses to compare proportions of these three genera as affected by season, datasets from May 2006 to 2007 were pooled (spring, *n* = 81) and datasets from October 2005, October 2006, to September 2007 were pooled (autumn, *n* = 149). The proportion of *Scytonema* TRFs was significantly higher in the autumn than in the spring. *Scytonema nifH* TRFs, when averaged across all treatments, comprised 82% of the T-RFLP profiles in the spring and 92% in the autumn (One way ANOVA, Wilcoxon/Kruskal–Wallace test *Z* = 0.000; Figure 4A). In contrast, the proportion of *Spirirestis* *nifH* TRFs was consistently higher in spring than autumn. *Spirirestis* *nifH* TRFs, when averaged across all treatments, comprised 17% of the T-RFLP profiles in the spring, and 3% in the autumn (One way ANOVA, Wilcoxon/Kruskal–Wallace test Z < 0.000; Figure 4A). *Nostoc* *nifH* TRFs, when averaged across all treatments, comprised 1% of the T-RFLP profiles in spring and 5% in autumn (Wilcoxon/Kruskal–Wallace test <0.000).

Terminal restriction fragment’s representing *Nostoc* *nifH* were detected sporadically (54% of all biocrust samples analyzed did not exhibit the *Nostoc*-specific 54 or 250 bp TRFs), and when detected, *Nostoc* TRFs typically comprised <5% of the total T-RFLP profile. Where *Nostoc* *nifH* peaks comprised >5% of the total T-RFLP profile (e.g., October 2007 LC) it was due to one to two samples in which the *Nostoc* TRFs comprised >70% of the total. In contrast to the filamentous form taken by *S. hyalinum* and *S. rafaelensis*, *N. commune* grows as macroscopic colonies, as photobionts of the lichen *C. tenax*, and as dense aseriate bundles or packets in biocrusts of the Colorado Plateau. Thus, it is not surprising that, in sporadic samples, *N. commune* TRFs comprised >70% of the total *nifH* profile.

The abundance of *Scytonema*, *Spirirestis*, and *Nostoc* *nifH* sequence types per g^−1^ of biocrust was estimated by multiplying the total *nifH* abundance g^−1^ soil (determined by qPCR) by the percent relative abundance of each genera-specific *nifH* sequence type (determined by T-RFLP). It is important to emphasize that the *nifH* abundances determined by this method should be treated as estimates only and are not meant as a definitive quantification of the *nifH* sequence types in the soils. T-RFLP is semi-quantitative, thus using TRF values to enumerate specific *nifH* sequence types from a total *nifH* population that was quantified by a separate method (qPCR) is a quasi-quantitative endeavor. Furthermore, a nested PCR with a total of 53 cycles and an intermediate dilution could likely bias toward dominant targets (i.e., *Scytonema* *nifH*; Suzuki and Giovannoni, 1996; Sipos et al., 2007). However, because these estimates were all derived in the same manner from highly replicated samples, they are useful as a *comparative* assessment of genera-specific *nifH* abundance in autumn and spring and over the time course of the study.

In aggregate, the abundance of heterocystous cyanobacteria increased steadily from October 2005 (1.6–3.4 × 10^6^ cells g^−1^ soil) to May 2007 (3.2–6.2 × 10^7^ cells g^−1^ soil) across all biocrust treatments. Over this time frame, *Scytonema* increased steadily, and regardless of season, from 1.4–3.2 × 10^6^ cells g^−1^ soil in October 2005 to 2.0–4.9 × 10^7^ cells g^−1^ soil in May 2007, an increase of ∼16-fold (Figure 4B). The numbers of *Spirirestis* and *Nostoc* increased over this time period also, but in a season-dependent fashion. Across treatments, there was an average of 3.2 × 10^5^ *Spirirestis* cells g^−1^ soil in October 2005, which increased to 2.7 × 10^6^ cells g^−1^ soil by May 2006, fell to 2.3 × 10^5^ cells g^−1^ soil over the summer of 2006 (September 2006 sampling date), rebounded to 1.2 × 10^7^ cells g^−1^ soil by May 2007, and was 2.4 × 10^4^ cells g^−1^ soil the following September (Figure 4B). Biocrusts sampled on October 2005, May 2006, and May 2007 contained ∼1 × 10^5^ *Nostoc* cells g^−1^ soil averaged across treatments, whereas those sampled on September 2006 and 2007 contained 1.4 × 10^6^ and 1.9 × 10^6^ *Nostoc* cells g^−1^ soil, respectively. However, because of the high spatial heterogeneity of the *Nostoc* T-RFLP profiles, these abundances should be treated as gross averages.

### T-RFLP analysis of biocrust *nifH* composition and relative abundance in response to increased temperature and FSVP

Comparisons of *Scytonema* and *Spirirestis* proportions across treatments revealed significant differences only from the first two sampling dates. *Spirirestis* *nifH* comprised a greater fraction of the total *nifH* pool in the LC biocrusts than in the IR, W, or IR&W samples collected in October 2005 and May 2006 (ANOVA *P* < 0.000; data not shown). *Scytonema* *nifH* comprised a lower fraction of the total *nifH* pool in the LC biocrusts compared to the other treatments during these sampling dates (ANOVA *P* < 0.000).

It was difficult to compare the proportion of *Nostoc* within the biocrust diazotrophic community across treatments because of the extreme spatial heterogeneity associated with the *Nostoc* T-RFLP profiles, and indeed statistical analysis did not reveal any significant differences. However, the *nifH* libraries from biocrusts collected in September 2007 that received FSVP (W, IR&W) consisted of <0.5% *Nostoc* sequences, whereas the *nifH* libraries from biocrusts receiving ambient precipitation (LC, IR) consisted of 5–10% *Nostoc* sequences.

## Discussion

Diazotrophic cyanobacteria in soil biocrusts are important contributors to C and N inputs in drylands, and an understanding of their population dynamics is needed to predict the impacts of environmental change on the ecosystem services that they provide. Here we examined the influence of season, increased temperature, and FSVP on the abundance and composition of the diazotrophic bacterial community within biocrusts of a semi-arid grassland of the Colorado Plateau. Strong seasonal shifts were observed in the relative proportions of the dominant diazotrophs (*Spirirestis*, *Scytonema*, and *Nostoc* spp.) in the biocrusts. Additionally, FSVP, but not increased year-round temperature (2–3°C), led to a decrease in total diazotroph abundance but no significant change in diazotroph community composition.

We did not find evidence for a distinct diazotrophic community in sub-biocrust soils (5 cm below the surface). Clone libraries and T-RFLP patterns indicated that *Spirirestis*, *Scytonema*, and *Nostoc* *nifH* sequences dominated in sub-biocrust soils, but were, on average sixfold less abundant than in the biocrusts. The TRF representing *nifH* Cluster O comprised <0.5% of the total T-RFLP profile in all biocrust and sub-biocrust (5 cm below the surface) samples analyzed. Cluster O TRFs were sporadically detected (∼5% of the T-RFLP profile) in soil samples retrieved 10 cm below the surface (data not shown). We were surprised to find such low levels of Custer O sequences at this study site, particularly in the sub-biocrust soils, as it was the dominant *nifH* sequence retrieved from the subsurface root zone and sub-biocrust soils (53 and 56%, respectively) of a nearby grassland with widespread biocrust cover (Yeager unpublished), and because we have found that it typically comprises 5–15% of the total *nifH* pool of biocrusts (e.g., Yeager et al., 2004). The potential role of the presumptive α-proteobacteria represented by Cluster O sequences or other bacteria in N_2_ fixation within and below the biocrusts remains to be determined, but these results further establish heterocystous cyanobacteria as the dominant diazotrophs in interplant soils of the Colorado Plateau.

Results from this study provide convincing evidence for temporal niche partitioning among the dominant diazotrophs in biocrusts at our study site, with *Scytonema* comprising a greater percentage of the community in the autumn and *Spirirestis* abundance peaking in the spring. We propose that *Scytonema* is better adapted for survival during the hot, dry summers of the Colorado Plateau than *Spirirestis*, resulting in a reduced representation of the latter in the diazotroph population during the autumn. A number of physiological, phylogenetic, and biogeographic observations support this conjecture, which we discuss below.

**Figure A2:**
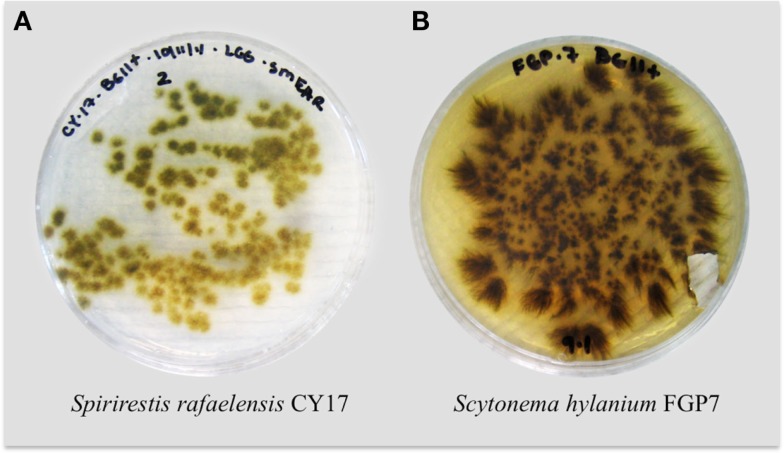
**Pigmentation of (A) *Spirirestis rafaelensis* and (B) *Scytonema hylanium* grown on BG11 medium agar plates**.

*Scytonema* is known for desiccation resistance and utilizes multiple survival mechanisms to cope with water deficiency, including survival structures, such as akinetes and hormogonia, thick polysaccharide sheaths, and osmoprotectants (sucrose, trehalose, glycine betaine, etc.; Tomaselli and Giovannetti, 1993; Gottlieb et al., 2005). We have qualitatively observed copious amounts of sheath material surrounding *Nostoc* and *Scytonema* in biocrusts, but far less associated with *Spirirestis* (data not shown). *Spirirestis* does form hormogonia, but akinetes have not been observed (Flechtner et al., 2002; Yeager et al., 2007). UV susceptibility could also differentiate *Spirirestis* from *Scytonema* in terms summer survival, since *Scytonema* appears to synthesize higher concentrations of the UV-protective pigments. We have consistently observed that *Scytonema* produces a greater quantity a yellow-brown pigment (scytonemin coloration) than *Spirirestis* when grown on agarized BG11 medium (Figure A2 in Appendix). Moreover, filaments of *Scytonema* and *Nostoc* in biocrusts are conspicuously more pigmented (orange/yellow-brown) than those of *Spirirestis* when viewed with a microscope (data not shown). Scytonemin is a UV-absorbing/screening pigment synthesized by a wide variety of terrestrial cyanobacteria and is crucial for the survival of these organisms when they are exposed to high intensities of incident light (Garcia-Pichel and Castenholz, 1991; Singh et al., 2010). In biocrusts of the Colorado Plateau, *Nostoc* and *Scytonema* spp. are known to produce scytonemin, and the concentrations of this sunscreen are highest in late summer/early autumn (Bowker et al., 2002).

Differences in the geographical distribution of *Scytonema* and *Spirirestis* also suggest that *Scytonema* may be more adept at survival in arid environments. We performed a BLAST analysis to reveal the geographical distribution of 16S rRNA gene sequences closely related (≥ 98% similarity) to those of *S. hyalinum* and *S. rafaelensis* (Table A1 in Appendix). Sequences closely related to both strains, particularly *S. rafaelensis*, have been identified from both cyanobacterial isolates and environmental samples obtained from soils of semi-arid and arid regions throughout the SW United States in hot (Mojave, Chihuahuan) and cool (Great Basin) deserts (Flechtner et al., 2002; Yeager et al., 2007; Berrendero et al., 2011). However, beyond the deserts of North America, 16S rRNA gene sequences closely related to *S. hyalinum* were scarce, reported by just 11 studies. *S. hyalinum-*related sequences are found in two of the driest environments on Earth, the Atacama Desert and the McMurdo Dry Valleys of Antarctica (Pointing et al., 2007; Azúa-Bustos et al., 2011; Lacap et al., 2011). Numerous *S. hyalinum-*related sequences were also found in biocrusts from the lowland, hot desert of Oman, and “fixed dunes” in an unnamed desert in China (Abed et al., 2010). Overall, the geographical distribution of *S. hyalinum-*related sequences indicates that *S. hyalinum* and closely related species have evolved to survive in environments where water is extremely scarce, UV exposure is high, and surface temperatures can exceed 50°C. Indeed, *S. hyalinum* belongs to the earliest branching clade arising after heterocyte-forming cyanobacteria purportedly evolved from a *Chroococcidiopsis*-like ancestor (Korelusová, 2008). *Chroococcidiopsis* are arguably the hardiest of the *Cyanobacteria*, able to survive extremes of desiccation and ionizing radiation, and are candidates for early colonization of land (Billi et al., 2011; Cockell et al., 2011).

On the other hand, we found no evidence that *S. rafaelensis* or closely related strains have evolved specifically for life in environments of extreme heat and/or water deprivation, but are rather capable of occupying a broad range of environments. Sequences that were ≥98% similar to the 16S rRNA gene of *S. rafaelensis* have been reported worldwide from biocrusts, agricultural soils, rice paddies, lakes, rivers, estuaries, etc. Furthermore, *S. rafaelensis* clusters with traditional members of the Nostocales, such as *Anabaena*, *Nostoc*, and *Tolypothrix*, which also inhabit a wide variety of environments (Korelusová, 2008).

In contrast to summertime, conditions in late winter/spring must be favorable for the proliferation of *Spirirestis* within biocrusts of the Colorado Plateau (see *Spirirestis* springtime rebound in Figure 4). During this period conditions for cyanobacterial growth can be optimal; snowmelt provides a steady source of moisture during the day, soil temperatures are often within the optimal range (20–25°C) for biocrust photosynthetic activity (Grote et al., 2010), and UV radiation is not at its summer peak. Conditions are also optimal for N_2_ fixation during the spring. In biocrusts of the Colorado Plateau, optimal N_2_ fixation typically occurs at air temperatures between 20 and 25°C, water contents between 20 and 60%, and during periods of active photosynthesis when light intensity is >200 μmol m^−2^ s^−1^ (Belnap, 2002, 2003; Belnap et al., 2004).

An obvious question arises from our results and the observed seasonal differences in N_2_ fixation: Are variations in N_2_ fixation rates of the Colorado Plateau biocrusts related to the seasonal oscillation of *Scytonema*/*Spirirestis* abundance? Different strains of heterocystous cyanobacteria exhibit varying N_2_ fixation rates. For example, it was recently shown that terrestrial strains of the *Tolypothrix* group (*Tolypothrix*, *Hassallia*, and *Scytonema*) typically exhibit higher rates of nitrogenase activity, as assessed by ethylene reduction, than *Nostoc* (Hrcková et al., 2010). The authors suggested that because *Nostoc* strains are typically surrounded by more mucilage, limited gas diffusion, and/or light penetration could lead to lower N_2_ fixation. Although *Spirirestis* and *Scytonema* are both filamentous in form, *Spirirestis* have much thinner sheaths than *Scytonema* (data not shown). Thus, it is possible that *Spirirestis* can fix N_2_ at higher rates under field-relevant conditions than *Scytonema*, and that *Spirirestis* contributes disproportionately to N input. Indeed, field measurements of daily integrated nitrogenase activity (acetylene reduction) *under optimal conditions* were shown to be ∼5- to 20-fold higher in the spring – when *Spirirestis* comprises a much larger fraction of the total diazotrophic community – than autumn (Housman et al., 2006). Although cyanobacterial biomass (including heterocystous spp.) in these biocrusts can vary between the spring and autumn, the differences are usually less than two- to three-fold (Bowker et al., 2002; this study). We did not measure N_2_ fixation rates for *Scytonema*, *Spirirestis*, or *Nostoc*, thus the contribution of these organisms to N input into semi-arid grasslands of the Colorado Plateau over seasons/years remains an open question.

In contrast to the large seasonal variation we observed in diazotroph community composition, experimentally manipulated changes to summer precipitation and year-round temperature (increases of 2-3°C) had little effect on the biocrust cyanobacterial community, including *Scytonema*, *Spirirestis*, and *Nostoc*, over the first 1.5 years of this field study (Johnson et al., 2012; this study). Across all treatments there was an increase in the total cyanobacterial 16S rRNA gene and *nifH* abundance between October 2005 and May 2007 (Figure 2A), and the results indicate that cyanobacterial population dynamics in biocrusts were regulated predominantly by ambient conditions for the site (as opposed to any plot-scale manipulations). We do not know why cyanobacterial abundance, including heterocystous spp., increased approximately one order of magnitude over this time frame. These results are similar to those of Cruz-Martinez et al. (2009), where it was reported that the microbial community composition in soils of a Northern California (USA) grassland responded to seasonal dynamics over the first 5 years of a manipulative field study, but not to the experimental treatments (three different watering regimes). The authors posited that microbial populations in soils of their Mediterranean-type grassland field site had evolved to endure moisture fluctuations and were thus resilient to the imposed precipitation patterns. During the sixth and seventh year of the study, treatment effects were noted, but only when the altered precipitation regime exacerbated prevailing climatic events. Furthermore, the responses were short-lived. The results from Cruz-Martinez et al. (2009) and those presented here underscore the importance of identifying the “normal operating range” (the naturally occurring amplitude of variation in a soil process/parameter) of soil microbial communities as described by Pereira e Silva et al. (2011). Without this underlying framework, our ability to predict, or even assess, the response, resiliency, and recovery of soil microbial communities in the face of environmental perturbations will be limited.

After the second summer of our field manipulations (September 2007), treatment effects did begin to emerge. There was a steep decrease (95% reduction) in both cyanobacterial 16S rRNA and *nifH* gene copies in biocrusts that had been subjected to FSVP. FSVP also led to a striking decline (∼25 to <2%) in moss (*S. caninervis*) ground cover at this study site, but after just a single summer treatment (Reed et al., 2012). It was determined that FSVP (≥1.2 mm but less than 5 mm) resulted in a negative C balance for *S. caninervis* (i.e., greater respiration than photosynthesis), and that the *S. caninervis* at this site could not withstand an increase in the frequency of these events for long before mortality occurred. Biocrust phototrophs are susceptible to damage from FSVP events in the summer (Lange et al., 1998; Belnap et al., 2004). When biocrusts are moist for brief periods (several hours or less), respiratory C loss exceeds photosynthetic C fixation, leading to a net C imbalance, and eventual mortality under harsh conditions (Jeffries et al., 1993; Lange et al., 1994, 1998; Belnap et al., 2004). This situation is compounded if precipitation events occur in the evening, when light is limited, or during periods of extreme heat when evaporation is rapid. We suspect that C imbalance was also the principal reason that the cyanobacterial population, including *Scytonema*, *Spirirestis*, and *Nostoc* spp., declined precipitously over the second summer of FSVP treatment. Unlike the Cruz-Martinez et al. (2009), where the effects of altered precipitation on the microbial population of a Mediterranean-type grassland were transitory, the biocrust community at our study site has yet to show signs of recovery during 5 years of continued FSVP (see Figure 7 in Johnson et al., 2012). These results demonstrate that it is not only the response/resiliency of microbial communities to climatic shifts that need to be understood, but also the extent to which microbial communities will recover in the face of enduring, altered climatic conditions.

Our data suggest that non-heterocystous cyanobacteria (e.g., *Microcoleus vaginatus*) were more prone to mortality from FSVP events, and possibly increased temperature, than were heterocystous species. FSVP events could preferentially lead to C imbalance in cyanobacteria spp. that must expend time and energy to reach the biocrust surface before the onset of photosynthesis. *M. vaginatus* and other non-heterocystous, filamentous cyanobacteria, which typically comprise the majority of the cyanobacterial biomass in biocrusts, are typically situated several mm below the biocrust surface to protect against UV damage (Garcia-Pichel and Belnap, 1996; Hu et al., 2003; Wu et al., 2011). It has been demonstrated that moisture triggers a phototaxic response in *M. vaginatus* (and other *Oscillatoria*), whereby it glides to the biocrust surface to engage in photosynthesis (Garcia-Pichel and Pringault, 2001). As the soil dries the cyanobacteria withdraw below the surface. This response occurs over a time frame of minutes to hours and requires substantial energy in the form of ATP prior to the onset of photosynthesis. Thus, due to the need for mobility, common non-heterocystous cyanobacteria such as *M. vaginatus* may expend more C and energy to prepare photosynthesis and be more negatively impacted if desiccation occurs quickly. In support of this idea, ecoregions that receive lower overall precipitation, but primarily in the form of summer rainfall, tend to have a greater proportion of heterocystous cyanobacteria than regions with high overall or winter precipitation patterns (Rosentreter and Belnap, 2003). On the other hand, Belnap et al. (2004) reported extensive decreases in quantum yield, chlorophyll *a*, and protective pigments in Colorado Plateau biocrusts containing a high proportion of *Scytonema* and *Nostoc* when treated with higher than average precipitation frequency spring thru autumn, but biocrusts dominated by *M. vaginatus* were much more resilient to the treatment. Based on a previous study (Bowker et al., 2002), it was suggested that cyanobacteria that depend on the synthesis of sunscreen pigments for photoprotection (i.e., *Nostoc* and *Scytonema*) quickly lose this capacity when facing C debt from FSVP events, and are consequently more susceptible to UV damage. From these studies, it is evident that heterocystous and non-heterocystous cyanobacteria in biocrusts can respond differently to FSVP events, but the underlying mechanism for these differences and the end results - in terms of ecosystem functioning – have not been established.

Taken together, the data presented here represent a significant step in understanding how a dryland biocrust diazotrophic community responds to changing climate, including seasonal changes or those resulting from global climate change. Because of our previous culturing efforts that linked biocrust *nifH* sequences to individual species (Yeager et al., 2007) and the relative simplicity of the biocrust diazotrophic community (>98% of the *nifH* sequences derived from either *Scytonema*, *Spirirestis*, or *Nostoc* spp.), we were able to conduct a comprehensive analysis at the genus/species level of taxonomic resolution. This presents a unique opportunity in that most molecular studies of free-living, soil diazotrophs have uncovered much greater levels of *nifH* richness (typically 10–100 *OTUs* per soil sample; Izquierdo and Nüsslein, 2006; Hsu and Buckley, 2008; Roesch et al., 2010; Pereira e Silva et al., 2011), and the genus/species of the microorganisms harboring many of the *nifH* sequence types was unknown. Having identified the three major diazotrophs in the biocrusts and seasonal patterns in their relative abundance, we can now begin to delve into the ecophysiology of these heterocystous cyanobacteria and into how ecophysiology and community composition relate to N dynamics of the Colorado Plateau in the face of changing climate.

## Conflict of Interest Statement

The authors declare that the research was conducted in the absence of any commercial or financial relationships that could be construed as a potential conflict of interest.

## Appendix

**Table A1:** **Environmental sources of *S. hyalinu**m*-related 16S rRNA gene sequences (outside drylands of North America)**.

Source	# Seqs^1^	% Similarity to *S. hyalinum*	16S rRNA copy^2^	bp^3^	Accession #^4^
Quartz hypolith, Yungay, Atacama Desert, Chile	5	99	1	1026	FJ891058
Quartz or sandstone endolith, McKelvey Valley, Antarctica	1	99	1	679	FJ490286
Biocrust, Oman	2	99	1	598	GU362257
Biocrust, “fixed dunes” desert, China	7	99–100	1	423	JN983731
Travertine, Yellowstone National Park, USA	1	100	1	378	AY790835
Citrus grove soil, FL, USA	1	99	1	398	GU589557
Quartz hypolith, near Antofagasta, Atacama Desert, Chile	1	99	1	379	FJ638282
Biocrust, Oman	28	98–100	2	747	GU362394
Hypolith, Australia	1	99	2	748	FJ230785
Wet wall, Manoa Valley, Hawaii	1	98	2	1063	HQ847565
Quartz hypoliths, worldwide	4	99	2	727	HM240897

*^1^Number of sequences closely related (≥98%) to *S. hyalinum* detected at the indicated site*.

*^2^*S. hyalinum* has two 16S rRNA gene copies that are ∼7% dissimilar that we have designated copy #1 (DQ531701) or #2 (DQ531704; Yeager et al., 2007)*.

*^3^Number of base pairs overlapping between the 16S rRNA gene (copy #1 or #2) from *S. hyalinum* and the sequence(s) from each source used to determine % similarity*.

*^4^Representative accession number for *S. hyalinum*-related 16S rRNA gene sequences from each source*.
